# Pan‐cancer molecular analysis of *EGFR* large fragment deletion in the Asian population

**DOI:** 10.1002/cam4.5603

**Published:** 2023-01-09

**Authors:** Jun Pu, Huannan Guo, Ruoying Yu, Qiuxiang Ou, Hua Bao, Xue Wu, Sanyuan Tang, Qingyong Chang

**Affiliations:** ^1^ Department of Neurosurgery Second Hospital of Kunming Medical University Kunming China; ^2^ Department of Medical Oncology General Hospital of Heilongjiang Province Land Reclamation Bureau Harbin People's Republic of China; ^3^ Nanjing Geneseeq Technology Inc. Nanjing China; ^4^ Department of Oncology The Affiliated Nanhua Hospital, Hengyang Medical School, University of South China Hengyang China; ^5^ Oncology Department The Second People's Hospital of Hunan Province Changsha China; ^6^ The Department of Neurosurgery Affiliated Zhongshan Hospital of Dalian University Dalian China

**Keywords:** colorectal cancer, comprehensive genome profiling, *EGFR* large fragment deletion, *EGFR*vIII, glioblastoma, lung cancer, melanoma

## Abstract

**Background:**

Large fragment deletion (LFD) of *EGFR* was associated with carcinogenesis in many types of cancers. However, the molecular features of *EGFR*‐LFD have not been studied in the Asian cancer population.

**Method:**

Here we retrospectively analyzed the targeted sequencing data from a large cancer database.

**Results:**

*EGFR*‐LFD was detected at a frequency of 0.03% with *EGFR*vIII being the most frequently observed LFD. *TERT*p variants were identified in 60% of the cases. *TP53* alterations (33%) were mutually exclusive with *TERT*p variants and coexisted with *EGFR*‐LFD in lung cancer and colorectal cancer. *EGFR* amplification (67%) and chromosome 10p deletion (53%) were the most focal‐level and arm‐level CNV in this cohort. *EGFR* exon2–17 skipping was found in the tumor tissue of one patient after progressing on osimertinib.

**Conclusion:**

Our study provided valuable insights into the distribution and molecular characteristics of *EGFR*‐LFD, hoping to shed light on the treatment management for *EGFR*‐LFD carriers.

## Graphical abstract

Novel EGF‐LFD identified in a large Asian cancer database.

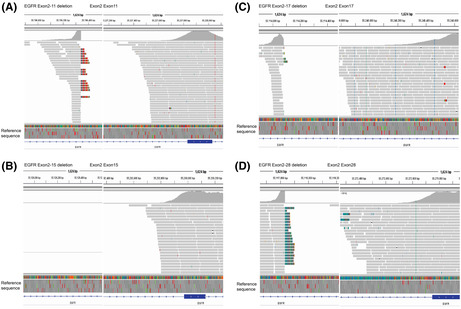

Abnormal overexpression and activation of epidermal growth factor receptor (EGFR) have been associated with various kinds of cancers. Large fragment deletion (LFD) is a type of structural variant that spans a large segment of human genes, contributing to carcinogenesis. Overall, there are five major types of *EGFR* large fragment deletion across the *EGFR* extracellular region and carboxyl‐terminal (C‐terminal) domain which were originally discovered in gliomas.^1^ *EGFR*vI and *EGFR*vII are two types of LFDs with N‐terminal domain deletion (exon1–13) and exon14–15 deletion, respectively. *EGFR*vII has been reported to associate with a growth advantage in tumor cells.^2^ *EGFR*vIII is the most common *EGFR* large fragment deletion identified in many malignancies, including glioblastoma,^3^ non‐small cell lung carcinoma,^4^ breast cancer,^5^ and ovarian carcinoma.^6^ The expression of *EGFR*vIII is restricted to cancer but not in normal tissue. *EGFR*vIII which is generated by gene rearrangement or abnormal mRNA splicing is characterized by an in‐frame deletion of exons2–7 of the EGFR coding sequence, producing a truncated 150‐kDa protein.^7^, ^8^ The lack of ligand‐binding region leads to the constitutive dimerization and activation of *EGFR*vIII.^9^ Interestingly, in glioblastoma, it has been reported that the presence of *EGFRvIII* is associated with *EGFR* gene amplification in most cases.^10^

E*GFR*vIV and *EGFR*vV are C‐terminal domain deletions that were reported to be sensitive to EGFR‐directed therapy.^11^ E*GFR*vIV and *EGFR*vIVa are associated with deletions of exon25–27 and *EGFR*vIVb consisted of exon25–26 deletion.^2^, ^12^ *EGFR* exon25–28 deletion is also termed *EGFR*vV. Computational analyses suggested that *EGFR*vIV and *EGFR*vV could be constitutively active due to the deletion of the auto‐inhibitory region.^13^ Other reported *EGFR*‐LFD includes deletions of *EGFR* exon2–5,^2^ exon12–13,^3^ exon4,^14^ exon27 and exon27–28,^11^ which have been classified as subtypes of *EGFR*vV. Here, we investigated the frequency and mutation spectrum of *EGFR‐LFD* using targeted sequencing in a large cohort of 66,500 Asian pan‐cancer cases. Novel *EGFR‐LFD*s and new cancer types with *EGFR‐LFD* have been identified.

In this study, we retrospectively reviewed records of 66,500 Asian cancer patients who underwent genetic testing using capture‐based targeted NGS between January 2014 to May 2020 at hospitals across China. The patient was considered as *EGFR* large fragment deletion positive if *EGFR* large fragment deletion was identified in the tumor tissue or plasma samples by targeted NGS. The NGS tests were performed in a centralized clinical testing center with a targeted gene panel according to the protocols reviewed. The mutation list of tumor tissue and plasma samples were shown in Table S1 and S2. The procedures in the study were approved by the ethics committee of the participating hospital and written informed consent was obtained from each patient for the use of their NGS results. Detailed sample collection information, methods of NGS sequencing and data analysis were shown in the Data S1.

The distribution of cancer types in this multi‐institutional database was shown in Figure S1. Lung cancer patients displayed the highest ratio, accounting for 66% (43,795 out of 66,500) of the total cases. The other frequently observed cancer types included colorectal cancer (10%, 6425 out of 66,500), gastric cancer (4%, 2729 out of 66,500), and breast cancer (3%, 2099 out of 66,500). There were 1612 cholangiocarcinoma, 145 glioblastoma, and 461 melanoma patients. The identified *EGFR*‐LFD was shown in Figure S1. Combining all the cancer types, *EGFR*‐LFD was identified at a rare ratio of 0.03% (17 out of 66,500). Among them, there were 9 males (53%), 7 females (41%) and one unknown gender with a median age of 54 years old, ranging from 42 to 85 years old (Table 1). *EGFR*‐LFD showed high frequency in glioblastoma, which was 5% (7 out of 145) of total glioblastoma cases. Other *EGFR*‐LFD cases included five cases of colorectal cancer, three cases of lung cancer, and one case of cholangiocarcinoma and melanoma (Figure S1; Table S3). *EGFR*vIII was identified in glioblastoma (7), colorectal cancer (4), and melanoma (1). Notably, novel *EGFR*‐LFDs were identified in unreported cancer types including deletions of *EGFR* exon2–11 (lung cancer), exon2–15 (lung cancer), exon2–17 (lung cancer, and colorectal cancer), exon2–28 (cholangiocarcinoma) (Figure S1). The next‐generation sequencing results of representative cases with novel *EGFR* large fragment deletion were shown in Figure 1.

**TABLE 1:** Cliopathological characteristics of patients with *EGFR* large fragment deletion

Characteristics	Cohort *n* = 17
Sex No.(Ratio)	
Male	9 (53%)
Female	7 (41%)
Unknown	1 (6%)
Age at diagnose	
Range	42–85 years old
Median	54 years old
Unknown	2
Histology No.(Ratio)	
Glioblastomas	7 (41%)
Colorectal cancer	5 (30%)
Lung cancer	3 (17%)
Cholangiocarcinoma	1 (6%)
Melanoma	1 (6%)
Large fragment deletion type No.(Ratio)	
Exon2–7 deletion	12 (70%)
Exon2–17 deletion	2 (12%)
Exon2–11 deletion	1 (6%)
Exon2–15 deletion	1 (6%)
Exon2–28 deletion	1 (6%)

**FIGURE 1:**
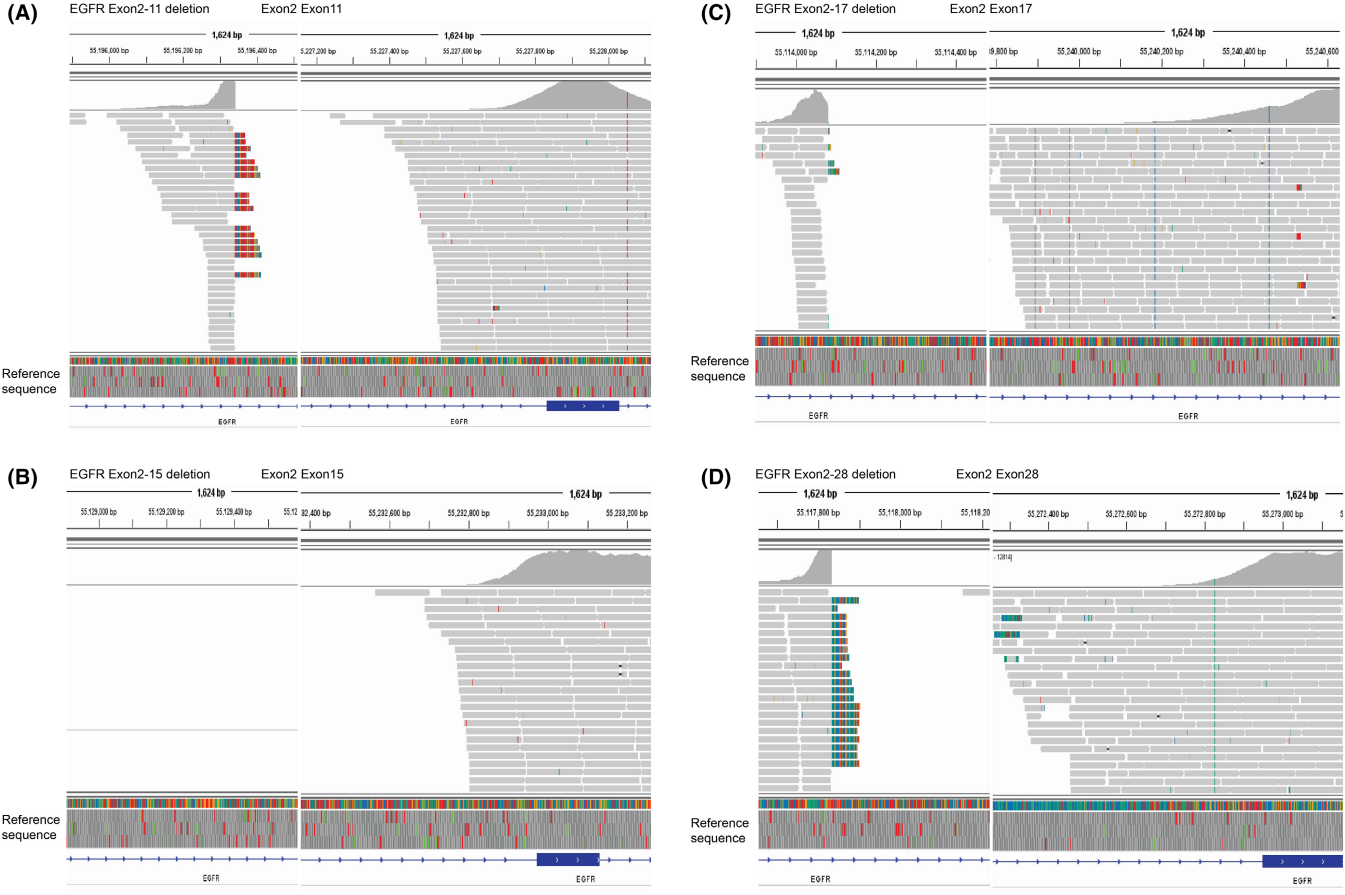
Visualization of novel *EGFR*‐LFD variants using the IGV browser. The bottom of each panel consists of the *EGFR* reference sequence shown. Different nucleotides were indicated by colors (T: red, A: green, C: blue, G: yellow). Mismatched bases were also indicated in the middle panel. LFD events included exon2–11 (A), exon2–15 (B), exon2–17(C) and exon2–28(D).

In total, tumor tissue was available in 15 patients and plasma was available in 6 patients. Using targeted sequencing, *EGFR*‐LFD was identified in tumor tissue of 13 patients (Figure 2) and plasma of 4 patients (Figure S2). The genomic landscape of *EGFR*‐LFD carriers in tumor tissue was in Figure 2. *TERT* promoter (*TERT*p) variants (60%) showed the highest frequency in *EGFR*‐LFD carriers. There were 6 glioblastoma cases with *TERT*p − 124C > T, one glioblastoma case with *TERT*p − 146C > T, and one melanoma case with *TERT*p − 146C > T. *TP53* alterations were found in 33% of the cases and were mutually exclusive with *TERT* alterations. Concurrent *TERT* alterations were only identified in *EGRF‐*LFD carriers with glioblastoma and melanoma while concurrent *TP53* alterations were only identified in *EGRF‐*LFD carriers with lung cancer and colorectal cancer. *EGFR* L858R was detected in the plasma of two patients (P12, P14 Figure S2). *KRAS* G12D and *EGFR* exon19 deletion was detected in the tumor tissue of P02 and P16, respectively. In consistence with the previous study,^15^ concurrent *EGFR* amplification was identified in 67% of the *EGF‐*LFD cohort including glioblastoma (6), colorectal cancer (3), and unknown (1). Over half of the *EGFR*‐LFD carriers harbored deletion on both arms of chromosome 10 (53%). And 12 out of 15 *EGRF‐*LFD patients were identified with multiple arm‐level CNVs in tumor tissue, suggesting these patients might have chromosome instability.

**FIGURE 2:**
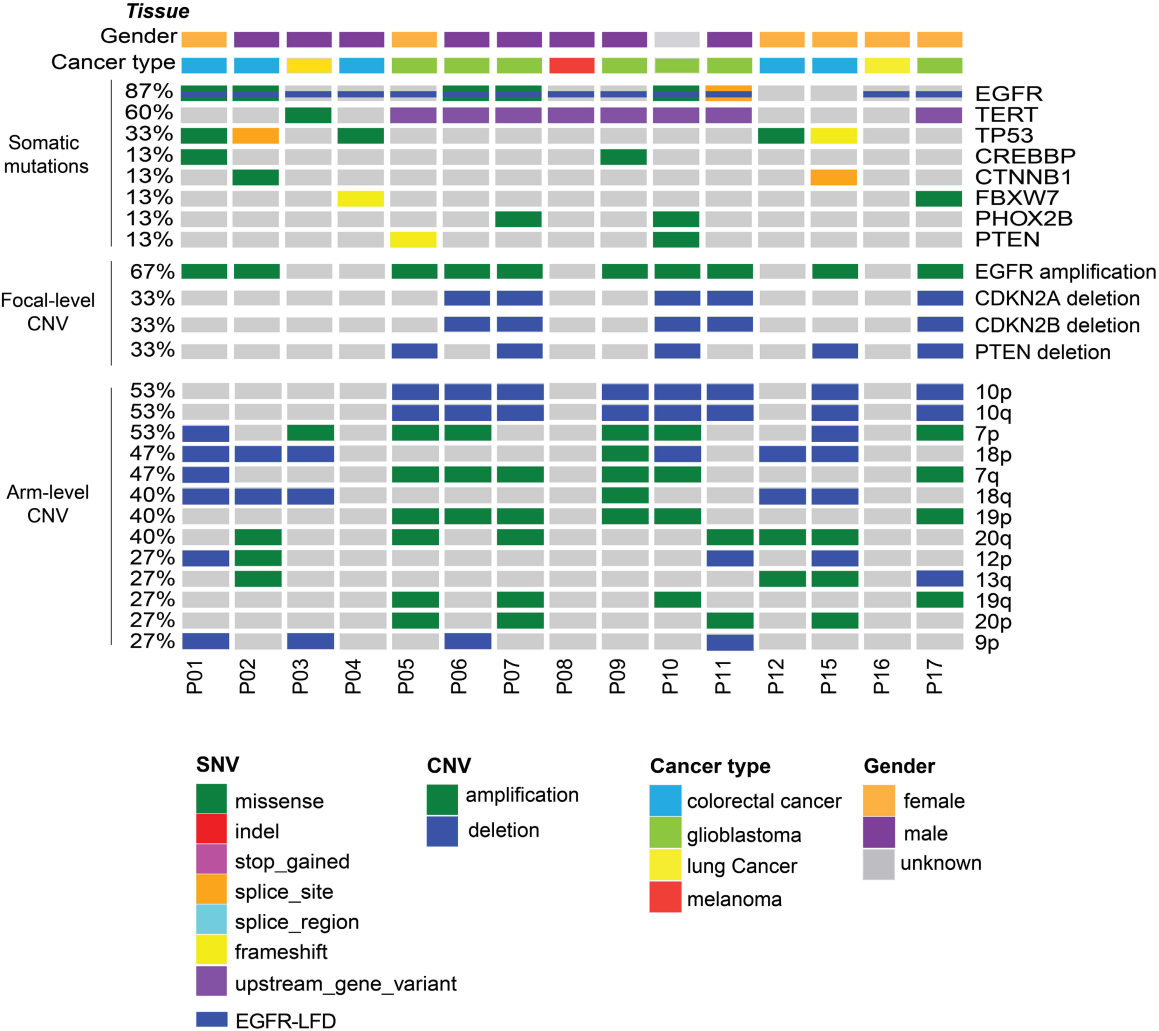
Genomic landscape of patients with *EGFR* large fragment deletion. Distribution of somatic alterations, focal‐level and arm level copy number variations in tumor tissue from patients with large fragment deletion. Each column represents one patient. Cancer type and gender were indicated at the top.

In this cohort, eight patients were treated with chemotherapy or chemoradiotherapy. EGFR‐targeted therapy was used in two patients while one patient received only surgery (Table S4). Three glioblastoma patients (P05, P07, and P17) with *EGFR* exon2–7 deletion, *EGFR* amplification, and *TERT* promoter variants in the brain tumor tissue received neoadjuvant or adjuvant chemoradiotherapy. Concurrent chemoradiotherapy was used in patient05 with *TERT* − 124C > T and achieved progression‐free survival (PFS) of 16 months. Patient07 was a male patient diagnosed with stage IV glioblastoma. He was first treated with radiotherapy and temozolomide for 2 months and then changed to temozolomide monotherapy for 8 months. The tumor slowly progressed after the treatment. Combinational treatment of temozolomide and apatinib was administrated later; however, the tumor progressed rapidly. He was identified with *TERT* − 146C > T and *EGFR* exon2–7 deletion after the sequential use of radiotherapy and chemotherapy. Interestingly, Patient16, a female patient with stage IV lung cancer, was treated with gefitinib and reached a progressive‐free survival (PFS) of 11 months. Later, osimertinib was used and achieved a PFS of 16 months. *EGFR* exon19 deletion (E746_A750del) and *EGFR* exon2–17 deletion were identified in the pleural tumor tissue after the patient progressed to Osimertinib. Osimertinib has been approved by FDA for treating non‐small cell lung cancer patients with *EGFR* exon19 deletion or exon21 (L858R) mutation.^16^ There was a possibility that *EGFR* exon2–17 deletion was associated with osimertinib‐resistant in this patient. More studies are warranted to validate our observation.

In conclusion, we reported on molecular characteristics of *EGFR*‐LFD in Asian pan‐cancer patients. Our analysis revealed that *EGFR*‐LFD accounted for 0.03% of all cancer cases, which was extremely rare. All *EGFR*‐LFD‐positive glioblastoma cases were with *EGFRvIII* and co‐occurred with *EGFR* amplification. Multiple studies have shown *EGFRvIII* and EGFR amplification was the most common genetic alteration in glioblastoma, which was in accord with this study.^15^, ^17^ Novel *EGFR*‐LFD including *EGFR* exon2–11, exon2–15, exon2–17, and exon2–28 deletion were identified. Meanwhile, *EGFR*‐LFD was identified in colorectal cancer and cholangiocarcinoma which, to the best of our knowledge, has not been reported. It seemed that the molecular features of *EGFR*‐LFD carriers were different among different types of cancer. Even though the *TP53* gene was also frequently altered in glioblastoma and melanoma,^18^, ^19^ in this cohort, *EGFR*‐LFD positive glioblastoma was only found with *TERT* alterations while *TP53* was usually concurrent with *EGFR*‐LFD in colorectal cancer, lung cancer.

The limitation of this study was the lack of the treatment history which prevented us from further analyzing the association between gene alterations and disease outcomes, especially in the cases with novel types of *EGFR*‐LFDs. Due to the retrospective nature of this study, we were unable to perform further assays such as IHC staining or RNA sequencing to validate the expression of the detected *EGFR*‐LFD in respective samples. However, previous studies have demonstrated a good performance of the same targeted panel as used in this study for detecting structural variance. Yao, Y et al. reported the *ALK* intergenic‐breakpoint rearrangement (IGR) detection in a large retrospective lung cancer cohort, and the authors have successfully validated the expression of all *ALK* rare IGRs using either RNA‐seq or IHC staining.^20^ Another study identified 27 known and novel *ALK* fusions which were all validated by either FISH or IHC staining.^21^ Nonetheless, further studies to validate the expression and functions of these novel *EGFR*‐LFDs are warranted.

In the lung cancer cell and mouse model, *EGFR*vIII were relatively resistant to gefitinib and erlotinib but sensitive to HKI‐272.^9^ In clinical settings, the efficacy of erlotinib and gefitinib in treating *EGFRvIII*+ glioblastoma could be further limited by the blood–brain barrier. Therefore, in other types of cancer with a brain tumor or possible brain metastasis such as glioblastoma and lung cancer, the existence of *EGFR*‐LFD might be taken into consideration during the EGFR‐targeted treatment management. Osimertinib, which displayed better penetration of the blood–brain barrier and high potency compared to other generation TKIs, might be a good choice for the *EGFR*‐LFD population.^22^ A preclinical study in cell lines showed that osimertinib inhibited the constitutive activity of *EGFR*vIII tyrosine kinase with high potency. On the other hand, a case report showed that the tumor in the right parietal lobe with *EGFR*vIII and *EGFR* amplification actively progressed on osimertinib while *EGFR*vIII‐negative left frontal lobe tumor reached a complete response.^23^, ^24^ *EGFR* amplification is associated with cancer aggressiveness and correlated with shortened overall survival in glioblastoma.^25^ It will be of great interest to study whether the *EGFR*‐LFD and concurrent *EGFR* amplification will contribute to the efficacy of EGFR TKIs in glioblastoma as well as the role of the novel *EGFR*‐LFDs in different cancer types.

## AUTHOR CONTRIBUTIONS

**Jun Pu:** Conceptualization (equal); data curation (equal); funding acquisition (equal). **Huannan Guo:** Conceptualization (equal); data curation (equal). **Ruoying Yu:** Formal analysis (equal); methodology (equal). **Qiuxiang Ou:** Writing – original draft (equal); writing – review and editing (equal). **Hua Bao:** Formal analysis (equal). **Xue Wu:** Writing – original draft (equal); writing – review and editing (equal). **Sanyuan Tang:** Conceptualization (equal); data curation (equal). **Qingyong Chang:** Conceptualization (equal); data curation (equal); investigation (equal).

## FUNDING INFORMATION

This study was supported by the project Supported by Basic Applied Research of Yunnan Province and Kunming Medical University 2018FE001(−172).

## CONFLICT OF INTEREST

Ruoying Yu, Qiuxiang Ou, Hua Bao, and Xue Wu are employees of Nanjing Geneseeq Technology Inc. The remaining authors have no conflicts of interest to declare.

## ETHICS STATEMENT

Patient consent form was obtained from each patient following the guideline of Institutional Review Board requirements and the Declaration of Helsink.

## Supporting information


Figure S1.
Figure S2.

Table S1.

Table S2.

Table S3.

Table S4.

Table S5.

Data S1.

## Data Availability

The data that support the findings of this study are available from the corresponding author upon reasonable request.
