# Supplementary material for: Pan‐cancer molecular analysis of EGFR large fragment deletion in the Asian population
Source: Cancer Med. 2023 Jan 9;12(7):8083–8. doi: 10.1002/cam4.5603 (PMC10134361; doi:10.1002/cam4.5603)

**Figure S1. The distribution of cancer type and *EGFR* large fragment deletion type.** (A) Distribution of different cancer type in the overall cohort. The number of cases in each cancer type was indicated in bracket. (B) Distribution of LFD and duplicated exons in each gene were shown in pie chart. The number of LFD cases was indicated in bracket.

**
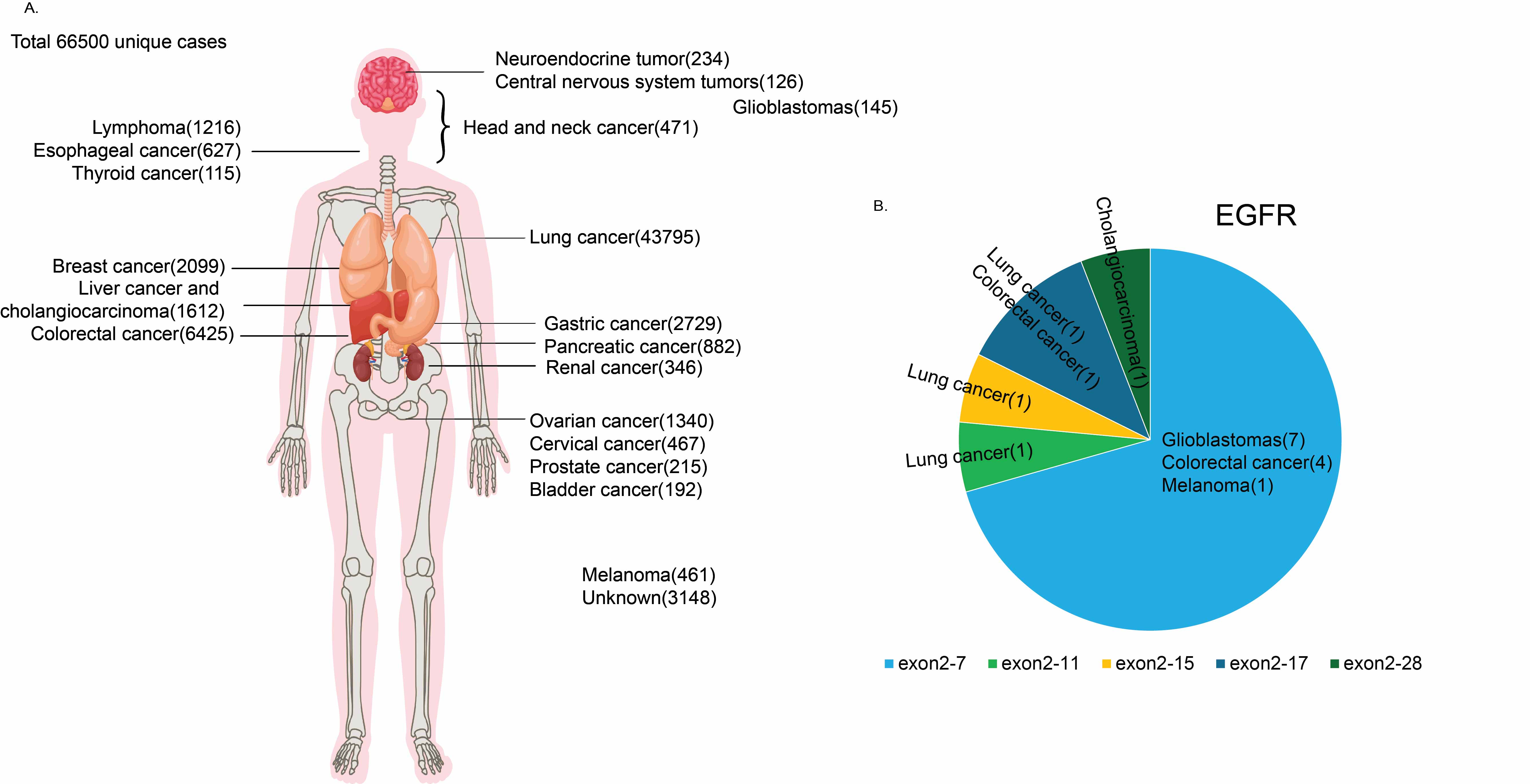
**

**Figure S2. Gene alterations in plasma of patients with *EGFR* large fragment deletion.** Each column represents one patient. The types of alterations were indicated by the color bar on the right.


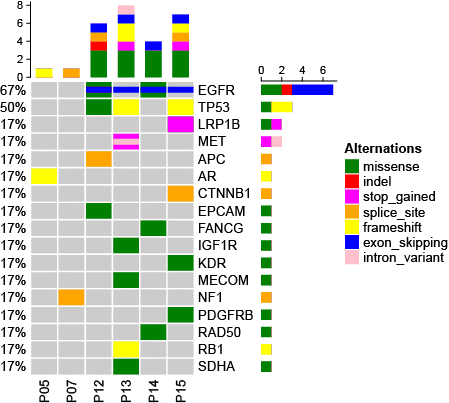

Supplement: Supplementary file 1 — Figure S1. Figure S2. [file CAM4-12-8083-s001.docx]
