# Supplementary material for: Pan‐cancer molecular analysis of EGFR large fragment deletion in the Asian population
Source: Cancer Med. 2023 Jan 9;12(7):8083–8. doi: 10.1002/cam4.5603 (PMC10134361; doi:10.1002/cam4.5603)
Supplement: Supplementary file 4 — Table S3. [file CAM4-12-8083-s005.docx]

**Table S3. Cliopathological characteristics of patients with *EGFR* large fragment deletion**

| Characteristics | Cohort n=17 |
| --- | --- |
| Sex No.(Ratio) | |
| Male | 9(53%) |
| Female | 7(41%) |
| Unknown | 1(6%) |
| Age at Diagnose | |
| Range | 42-85 years old |
| Median | 54 years old |
| Unknown | 2 |
| Histology No.(Ratio) | |
| Glioblastomas | 7(41%) |
| Colorectal cancer | 5(30%) |
| Lung cancer | 3(17%) |
| Cholangiocarcinoma | 1(6%) |
| Melanoma | 1(6%) |
| Large fragment deletion type | **No.(Ratio)** |
| Exon2-7 deletion | 12(70%) |
| Exon2-17 deletion | 2(12%) |
| Exon2-11 deletion | 1(6%) |
| Exon2-15 deletion | 1(6%) |
| Exon2-28 deletion | 1(6%) |
