# Supplementary material for: Pan‐cancer molecular analysis of EGFR large fragment deletion in the Asian population
Source: Cancer Med. 2023 Jan 9;12(7):8083–8. doi: 10.1002/cam4.5603 (PMC10134361; doi:10.1002/cam4.5603)
Supplement: Supplementary file 6 — Table S5. [file CAM4-12-8083-s004.docx]

Table S5. List of 425 genes covered by GeneseeqPrime

| ABCB1 | BMPR1A | CTCF | ERBB4 | FRG1 | JAK3 | MLLT4 | PBRM1 | PTPN13 | SMAD2 | TP53 |
| --- | --- | --- | --- | --- | --- | --- | --- | --- | --- | --- |
| ABCC2 | BRAF | CTLA4 | ERCC1 | GATA1 | JARID2 | MPL | PDCD1 | QKI | SMAD3 | TP63 |
| ADH1B | BRCA1 | CTNNB1 | ERCC2 | GATA2 | JUN | MRE11A | PDCD1LG2 | RAC1 | SMAD4 | TPMT |
| AIP | BRCA2 | CUL3 | ERCC3 | GATA3 | KDM5A | MSH2 | PDE11A | RAC3 | SMAD7 | TSC1 |
| AKT1 | BRD4 | CUX1 | ERCC4 | GATA4 | KDR | MSH6 | PDGFRA | RAD50 | SMARCA4 | TSC2 |
| AKT2 | BRIP1 | CXCR4 | ERCC5 | GATA6 | KEAP1 | MTHFR | PDGFRB | RAD51 | SMARCB1 | TSHR |
| AKT3 | BTG2 | CYLD | ESR1 | GNA11 | KIF1B | MTOR | PDK1 | RAD51B | SMO | TTF1 |
| ALDH2 | BTK | CYP19A1 | ETV1 | GNAQ | KIT | MUTYH | PGR | RAD51C | SOCS1 | TUBB3 |
| ALK | BUB1B | CYP2A13 | ETV4 | GNAS | KITLG | MYC | PHOX2B | RAD51D | SOS1 | TYMS |
| AMER1 | EMSY | CYP2A6 | ETV5 | GRIN2A | KLLN | MYCL | PIK3C3 | RAD54L | SOX2 | U2AF1 |
| APC | CASP8 | CYP2A7 | ETV6 | GRM3 | KMT2A | MYCN | PIK3CA | RAF1 | SPOP | UGT1A1 |
| AR | CBL | CYP2B6 | EWSR1 | GRM8 | KMT2B | MYD88 | PIK3CD | RARA | SPRED1 | VAMP2 |
| ARAF | CBLB | CYP2C19 | EXT1 | GSTM1 | KMT2C | MYH9 | PIK3R1 | RARG | SPRY4 | VEGFA |
| ARID1A | CCND1 | CYP2C9 | EXT2 | GSTM4 | KMT2D | NAT1 | PIK3R2 | RASGEF1A | SRC | VHL |
| ARID1B | CCNE1 | CYP2D6 | EZH2 | GSTP1 | KRAS | NBN | PKHD1 | RB1 | SRSF2 | WAS |
| ARID2 | CD274 | CYP3A4 | EZR | GSTT1 | LHCGR | NCOR1 | PLAG1 | RECQL4 | SRY | WISP3 |
| ARID5B | CD74 | CYP3A5 | FANCA | HDAC2 | LMO1 | NF1 | PLCB4 | RELN | STAG2 | WRN |
| ASCL4 | CDA | CYSLTR2 | FANCC | HDAC9 | LRP1B | NF2 | PLK1 | RET | STAT3 | WT1 |
| ASXL1 | CDC73 | DAXX | FANCD2 | HGF | LYN | NFE2L2 | PMS1 | RHOA | STK11 | XPA |
| ATF1 | CDH1 | DDR2 | FANCE | HLA-A | LZTR1 | NFKBIA | PMS2 | RICTOR | STMN1 | XPC |
| ATIC | CDK10 | DENND1A | FANCF | HNF1A | MAP2K1 | NKX2-1 | POLD1 | RNF43 | SUFU | XRCC1 |
| ATM | CDK12 | DHFR | FANCG | HNF1B | MAP2K2 | NOTCH1 | POLD3 | ROS1 | TACC3 | XRCC2 |
| ATR | CDK4 | DICER1 | FANCI | HRAS | MAP2K4 | NOTCH2 | POLE | RPTOR | TAP1 | YAP1 |
| ATRX | CDK6 | DLL3 | FANCL | IDH1 | MAP3K1 | NOTCH3 | POLH | RRM1 | TAP2 | ZNF217 |
| AURKA | CDK8 | DNMT3A | FANCM | IDH2 | MAP3K4 | NPM1 | POT1 | RUNX1 | TEK | ZNF703 |
| AURKB | CDKN1A | DOT1L | FAT1 | IFNA6 | MAX | NQO1 | PPARD | RUNX1T1 | TEKT4 |  |
| AXIN2 | CDKN1B | DPYD | FBXW7 | IFNB1 | MCL1 | NRAS | PPP2R1A | SBDS | TERC |  |
| AXL | CDKN1C | DTL | FGF19 | IFNE | MDM2 | NRG1 | PRDM1 | SDC4 | TERT |  |
| B2M | CDKN2A | DUSP2 | FGFR1 | IFNG | MDM4 | NSD1 | PREX2 | SDHA | TET2 |  |
| BAD | CDKN2B | EGFR | FGFR2 | IFNGR1 | MECOM | NTRK1 | PRF1 | SDHB | TGFBR2 |  |
| BAI3 | CDKN2C | EIF1AX | FGFR3 | IFNGR2 | MED12 | NTRK2 | PRKACA | SDHC | THADA |  |
| BAK1 | CEBPA | EP300 | FGFR4 | IGF1R | MEF2B | NTRK3 | PRKAR1A | SDHD | TMEM127 |  |
| BAP1 | CEP57 | EPAS1 | FH | IGF2 | MEN1 | NUTM1 | PRKCI | SEPT9 | TMPRSS2 |  |
| BARD1 | CHD4 | EPCAM | FLCN | IKBKE | MET | PAK3 | PRKDC | SETBP1 | TNFAIP3 |  |
| BAX | CHD8 | EPHA2 | FLT1 | IKZF1 | MGMT | PALB2 | PRSS1 | SETD2 | TNFRSF11A |  |
| BCL2 | CHEK1 | EPHA3 | FLT3 | IL7R | MITF | PALLD | PRSS3 | SF3B1 | TNFRSF14 |  |
| BCL2L11 | CHEK2 | EPHA5 | FLT4 | INPP4B | MLH1 | PARK2 | PTCH1 | SGK1 | TNFRSF19 |  |
| BCR | CREBBP | ERBB2 | FOXA1 | IRF2 | MLH3 | PARP1 | PTEN | SKP2 | TNFSF11 |  |
| BIRC3 | CRKL | ERBB2IP | FOXL2 | JAK1 | MLLT1 | PARP2 | PTK2 | SLC34A2 | TOP1 |  |
| BLM | CSF1R | ERBB3 | FOXP1 | JAK2 | MLLT3 | PAX5 | PTPN11 | SLC3A2 | TOP2A |  |

List of 43 genes with selected intron, or UTR regions covered

| ALK | CD74 | FGFR1 | NOTCH2 | RET | TERC |
| --- | --- | --- | --- | --- | --- |
| BCL2 | EGFR | FGFR2 | NTRK1 | ROS1 | TERT |
| BCR | ETV1 | FGFR3 | NTRK2 | SDC4 | RB1 |
| BIRC3 | ETV4 | FGFR4 | NTRK3 | SLC34A2 |  |
| BRAF | ETV5 | KIT | NUTM1 | SLC3A2 |  |
| BRCA1 | ETV6 | KMT2A | PDGFRA | TACC3 |  |
| BRCA2 | EWSR1 | MET | RAF1 | TMPRSS2 |  |
| BRD4 | EZR | MYC | RARA | VAMP2 |  |
