# Supplementary material for: Pan‐cancer molecular analysis of EGFR large fragment deletion in the Asian population
Source: Cancer Med. 2023 Jan 9;12(7):8083–8. doi: 10.1002/cam4.5603 (PMC10134361; doi:10.1002/cam4.5603)
Supplement: Supplementary file 7 — Data S1. [file CAM4-12-8083-s007.docx]

**Supplementary Methods**

**Sample collection**

5 to 10 mL peripheral blood was collected from each patient in EDTA-coated tubes (BD Biosciences). Plasma was extracted within 2 hours of blood collection and shipped to the central testing laboratory within 48 hours. Formalin-fixed paraffin-embedded (FFPE) tumor tissue blocks/sections or fresh tumor tissues were obtained from the hospitals, with confirmation by the pathologists for diagnosis and tumor purity. The targeted sequencing was performed with GeneseeqPrime panel of 425 genes (Geneseeq Technology Inc.), which covers all EGFR exons (Table S5).

**DNA extraction and quantification, library preparation**

The DNA extraction, quantification, and library preparation were performed as previously described[1]. In brief, FFPE samples were de-paraffinized with xylene, and DNA was extracted using the QIAamp DNA FFPE Tissue Kit (Qiagen) according to the manufacturer’s protocols. Genomic DNA from fresh tumor tissue was extracted using the DNeasy Blood & Tissue Kit (Qiagen) according to the manufacturer's protocols. Peripheral blood samples were centrifuged at 1800g for 10min. Then the plasma was isolated for extraction of cfDNA and the genomic DNA of white blood cells in sediments were served as normal controls. A circulating nucleic acid kit (Qiagen, Germany) was used to purify cfDNA from plasma. The genomic DNA from white blood cells was extracted using DNeasy Blood and Tissue Kit (Qiagen). Genomic DNA was qualified using a Nanodrop2000 (Thermo Fisher Scientific, Waltham, MA), and cfDNA fragment distribution was analyzed on a Bioanalyzer 2100 using the High Sensitivity DNA Kit (Agilent Technologies, Santa Clara, CA). All DNA was quantified using the dsDNA HS assay kit on a Qubit 3.0 fluorometer (Life Technology, US) according to the manufacturer’s recommendations. Sequencing libraries were prepared using the KAPA Hyper Prep kit (KAPA Biosystems) with an optimized manufacturer’s protocol. cfDNA or fragmented genomic DNA underwent end-repairing, A-tailing and ligation with indexed adapters, followed by size selection using Agencourt AMPure XP beads (Beckman Coulter). Target enrichment was achieved with customized xGen lockdown probes (Integrated DNA Technologies). The mean sequencing depths of tumor tissue and plasma were 500X and 3000X respectively.

**Data processing**

Sequencing data were processed as previously described[1]. The data was demultiplexed and underwent FASTQ file quality control using Trimmomatic to remove low quality data (below 15) or N bases[2]. Qualified reads were mapped to the reference human genome hg19 using Burrows-Wheller Aligner (BWA-mem, v0.7.12; <https://github.com/lh3/bwa/tree/master/bwakit>). Genome Analysis Toolkit (GATK 3.4.0;  <https://software.broadinstitute.org/gatk/>) was used for base quality score recalibration and local realignment around indels. PCR duplicates were remove using Picard. VarScan2 was used for the detection of insertion/deletion mutations and single-nucleotide variations (SNVs) [3]. Calls with at least 0.2% mutant allele frequency (MAF) of somatic variant and with at least three supporting-reads from both directions were kept. Common SNVs were excluded with following criteria: 1) present in >1% population in the 1000 Genomes Project or 2) present in the Exome Aggregation Consortium (ExAC) 65,000 exomes database. The resulting mutation list was then filtered with an in-house list of recurrent artifacts from a normal pool of whole blood samples. Sequencing of matched white blood cells from each patient was performed to further eliminate germline variants, sequencing artifacts, clonal hematopoiesis. The Copy number alterations (CNVs) were analyzed by CNVkit and GISTIC algorithm as previously described[4]. Fusion And Chromosomal Translocation Enumeration and Recovery Algorithm (FACTERA) was applied to structural variants(SV) detection using default parameters[5]. FACTERA is designed for the detection of breakpoints in targeted sequencing data and can be applied to any BAM file with paired-end reads. The SV reads called were manually reviewed and conﬁrmed on Integrative Genomics Viewer (IGV). Tumor mutational burden (TMB) was calculated based on the number of somatic base substitutions and indels in the targeted regions covering 0.85 Mb of coding genome, excluding known driver mutations as they are over-represented in the panel.

Reference:

1. Yang, Z., et al., *Investigating Novel Resistance Mechanisms to Third-Generation EGFR Tyrosine Kinase Inhibitor Osimertinib in Non-Small Cell Lung Cancer Patients.* Clin Cancer Res, 2018. **24**(13): p. 3097-3107.

2. Bolger, A.M., M. Lohse, and B. Usadel, *Trimmomatic: a flexible trimmer for Illumina sequence data.* Bioinformatics, 2014. **30**(15): p. 2114-20.

3. Koboldt, D.C., et al., *VarScan 2: somatic mutation and copy number alteration discovery in cancer by exome sequencing.* Genome Res, 2012. **22**(3): p. 568-76.

4. Zhu, C., et al., *Genomic Profiling Reveals the Molecular Landscape of Gastrointestinal Tract Cancers in Chinese Patients.* Front Genet, 2021. **12**: p. 608742.

5. Newman, A.M., et al., *FACTERA: a practical method for the discovery of genomic rearrangements at breakpoint resolution.* Bioinformatics, 2014. **30**(23): p. 3390-3.
